# Identification of CCND1 and IL7R as core JAK-STAT pathway genes promoting hepatitis B-related liver fibrosis

**DOI:** 10.3389/fimmu.2026.1776431

**Published:** 2026-03-23

**Authors:** Jiahao Wu, Chen Hu, Guang Yang

**Affiliations:** 1 Guangzhou Twelfth People’s Hospital, Guangzhou, China; 2 Guangzhou Occupational Disease Prevention and Treatment Hospital, Guangzhou, China; 3 The Affiliated Guangzhou Twelfth People’s Hospital, Guangzhou Medical University, Guangzhou, China

**Keywords:** HBV, JAK-STAT, liver fibrosis, machine learning, WGCNA

## Abstract

**Background:**

Liver fibrosis is one of the most common complications in patients with HBV infection. Characterized by excessive extracellular matrix deposition and hepatic stellate cell activation, it is tightly linked to the JAK-STAT pathway that regulates inflammatory and fibrogenic processes.

**Methods:**

The GSE84044 dataset was retrieved from the GEO database. After grouping and preprocessing, DEGs were screened with the limma package and then subjected to functional enrichment analysis. WGCNA was performed to identify fibrosis-related module genes. Candidate key genes were obtained by intersecting these WGCNA module genes with the DEGs and JAK-STAT genes. A diagnostic model was constructed via machine learning algorithms to further filter core genes. Subsequently, GSEA, GSVA and immune infiltration analysis were conducted to explore the biological functions of these core genes. Mendelian randomization (MR) was employed to verify the causal relationships among the target genes. Furthermore, the miRNA-mRNA-TF network of the core genes were constructed. ScRNA analysis was performed to validated our finding. Finally, the expression levels of core genes were experimentally validated by Western blot and qPCR.

**Results:**

CCND1 and IL7R were identified as hub genes of the JAK-STAT pathway through integrated analyses. Both genes are significantly upregulated and exert synergistic effects in HBV-related liver fibrosis, with the constructed diagnostic model achieving an AUC of 0.890. Functional enrichment indicated their involvement in immune regulatory and fibrotic pathways, while immune infiltration analysis revealed a close association with M1 macrophages and other immune cell subsets. MR analysis confirmed a significant positive causal effect of IL7R on liver fibrosis. The miRNA-mRNA-TF regulatory network highlighted their post-transcriptional and transcriptional regulatory mechanisms. scRNA-seq validated cell-type-specific expression patterns of CCND1 (epithelial cells, endothelial cells, hepatocytes, macrophages) and IL7R (T/NK cells). Western blot and qPCR further confirmed the upregulation of these genes in HBV-related fibrotic liver tissues at both protein and mRNA levels.

**Conclusions:**

In summary, CCND1 and IL7R are core JAK-STAT pathway genes associated with HBV-related liver fibrosis, with IL7R showing a significant causal role. They regulate immune microenvironment and may serve as diagnostic biomarkers.

## 1 Introduction

Hepatitis B virus (HBV) infection remains a significant global health challenge, affecting approximately 254 million individuals worldwide, with nearly 1.2 million new infections occurring annually (1). Despite substantial progress in vaccination programs and antiviral therapies, HBV continues to impose a substantial burden on healthcare systems, particularly in developing regions (2, 3). The natural progression of chronic hepatitis B is characterized by a spectrum of hepatic complications, among which liver fibrosis represents a critical pathological milestone (4). Hepatic fibrosis, defined as the excessive accumulation of extracellular matrix proteins including collagen types I and III, serves as a wound-healing response to chronic liver injury. In the context of HBV infection, this process becomes dysregulated and self-perpetuating, leading to progressive architectural distortion of hepatic tissue (5). Notably, HBV-related fibrosis exhibits unique characteristics that distinguish it from other etiologies, including a more rapid progression rate and limited reversibility. The virus-encoded X protein (HBx) plays a pivotal role in this process by directly activating hepatic stellate cells (HSCs) and enhancing the expression of profibrotic mediators such as transforming growth factor-β (TGF-β) and α-smooth muscle actin (6). After hepatic fibrosis has become established in patients with chronic hepatitis B, it exhibits limited responsiveness to conventional antiviral therapies; approximately 20–30% of these individuals continue to progress to cirrhosis despite adequate viral suppression. This irreversible progression underscores the urgent need for therapeutic strategies that target the fundamental molecular mechanisms underlying fibrogenesis (7).

The pathogenesis of HBV-related liver fibrosis involves a complex interplay between viral factors, host immune responses, and molecular signaling cascades. At the cellular level, activated HSCs serve as the primary effector cells, undergoing transdifferentiation into myofibroblast-like cells that secrete excessive extracellular matrix components. This activation process is orchestrated by a network of cytokines, growth factors, and inflammatory mediators, creating a profibrotic microenvironment that perpetuates tissue injury (8–10). The clinical significance of hepatic fibrosis extends beyond structural alterations, as it disrupts normal hepatic function, impairs blood flow, and creates a premalignant environment that significantly increases the risk of hepatocellular carcinoma development (11). The molecular mechanisms underlying HBV-induced fibrogenesis involve multiple interconnected pathways, including oxidative stress, endoplasmic reticulum stress, and immune-mediated injury. Recent evidence has highlighted the crucial role of specific signaling cascades in coordinating these diverse pathological processes, with the Janus kinase-signal transducer and activator of transcription (JAK-STAT) pathway emerging as a central regulator of fibrotic progression (12, 13).

The JAK-STAT signaling pathway represents a fundamental mechanism for cytokine-mediated signal transduction, comprising four JAK family members (JAK1, JAK2, JAK3, and TYK2) and seven STAT proteins (STAT1–6 and STAT5A/B) (14, 15). This pathway is activated by various inflammatory cytokines, including interleukin-6 (IL-6), interferon-γ, and growth factors, which are abundantly expressed in the fibrotic liver microenvironment. Upon ligand binding, JAKs undergo autophosphorylation and subsequently phosphorylate STAT proteins, leading to their nuclear translocation and activation of target gene transcription (16). In the context of hepatic fibrosis, JAK-STAT signaling serves multiple pathological functions, including promoting HSC activation, stimulating collagen synthesis, and modulating inflammatory responses. Recent studies have demonstrated that STAT3, in particular, plays a dual role in hepatocellular injury and fibrogenesis, with its activation correlating directly with fibrosis severity in chronic hepatitis B patients (12, 17). The therapeutic potential of targeting JAK-STAT signaling has been validated by preclinical studies showing that pharmacological inhibition of JAK2 significantly reduces liver fibrosis in experimental models, decreasing collagen deposition and HSC activation markers (18). In addition, there is a lack of research on specific genes associated with HBV-related hepatic fibrosis in the JAK-STAT pathway; these genes are not only promising to serve as potential biomarkers for fibrosis progression, but also can be developed as therapeutic targets for precision medicine strategies.

In this study, we employs comprehensive bioinformatics approaches to identify critical JAK-STAT pathway genes that drive HBV-related hepatic fibrogenesis. By integrating multi-omics data and advanced computational analyses, we have pinpointed key molecular signatures that distinguish fibrotic progression in hepatitis B patients. These findings provide novel insights into the molecular mechanisms underlying HBV-induced liver fibrosis and identify potential therapeutic targets for developing precision medicine strategies aimed at preventing and reversing fibrotic progression in chronic hepatitis B patients.

## 2 Methods

### 2.1 Data downloading and processing

We downloaded the transcriptomic data and clinical information for the hepatitis B-related liver fibrosis dataset GSE84044 from the Gene Expression Omnibus (GEO) database (https://www.ncbi.nlm.nih.gov/gds/). Based on the Scheuer score (s) classification in the clinical information file, samples with a score of 0 were classified as the non-fiber group (Control, n = 43), while those with scores > 0 were assigned to the fiber group (Fibre, n = 81). The transcriptomic data was log2-transformed by the “limma” package. The JAK-STAT pathway related genes were downloaded from the MSigDB database (https://www.gsea-msigdb.org/). The outcome dataset of the Mendelian Randomization (MR) analysis was downloaded from the R12 FinnGen database (https://www.finngen.fi/en/access_results). Finally, we constructed a miRNA-mRNA-TF interaction network based on the core genes of JAK-STAT pathway.

### 2.2 Identify the differentially expressed genes and functional analysis

“limma” package was used to screen for differentially expressed genes (DEGs) in hepatitis B-related liver fibrosis (GSE84044), with the filtering criteria of adjusted P-value ≤ 0.05 and |log2 fold change (FC)| ≥ 0.5. “ggplot2” and “pheatmap” packages were utilized to generate volcano plots and gene clustering heatmaps of the DEGs. “clusterProfiler” package was employed to perform Gene Ontology (GO) and Kyoto Encyclopedia of Genes and Genomes (KEGG) analyses on the DEGs. Subsequently, the “enrichplot” package was used to explore their biological functions and signaling pathways, and “ggplot2” is applied to visualize the results.

### 2.3 Weighted gene co-expression network analysis

The “WGCNA” package was employed to identify co-expression gene modules with high biological significance and explore the relationships between gene networks and diseases (19). Firstly, different β parameters were tested to construct a scale-free network, with an appropriate soft-thresholding power set. Ultimately, β = 13 was selected as the soft threshold. Secondly, the dynamic tree cutting algorithm was adopted, with each module required to contain a minimum of 100 genes. Subsequently, the dynamic hybrid merging method was utilized to merge similar modules using a threshold of 0.2. Finally, Pearson correlation coefficients between the module eigengenes (MEs) of each module and sample traits were calculated to screen for trait-related modules. The “VENN” package was used to intersect differentially expressed genes (DEGs), WGCNA module genes, and JAK-STAT pathway genes, thereby initially identifying the preliminarily selected JAK-STAT genes. Finally, the “clusterProfiler” package was applied to perform KEGG and GO enrichment analyses on these intersected genes, with visualization conducted using “ggplot2”.

### 2.4 Identify the JAK-STAT core genes and construct a diagnostic model

The “Caret” package was performed to divided the Control and Fibre sample as a ratio of 7: 3 into a training cohort and test cohort. We employed a machine learning framework to assess the diagnostic value of the preliminarily selected JAK-STAT genes. Firstly, we standardized the gene expression data for both the training and validation sets, and then constructed a machine learning algorithm model integrating 113 combinations.These algorithms encompass the Least Absolute Shrinkage and Selection Operator (LASSO), Ridge regression, Elastic Net, Support Vector Machine (SVM), Generalized Linear Model Boosting (glmBoost), Partial Least Squares Generalized Linear Model (plsRglm), Stepwise Generalized Linear Model (StepGLM), Random Forest (RF), Gradient Boosting Machine (GBM), Linear Discriminant Analysis (LDA), eXtreme Gradient Boosting (XGBoost), and Naïve Bayes. Subsequently, we selected the best combination as our diagnostic model based on the AUC value. We evaluated model performance using the area under the receiver operating characteristic(ROC) curve and constructed a confusion matrix to assess its accuracy. We performed differential analysis of the model genes in the GSE84044 dataset, displayed the results with boxplots, and calculated Pearson correlation coefficients to quantify relationships among the JAK-STAT core genes. Individual diagnostic performance of each core gene was evaluated with ROC curves. Finally, a co-expression network illustrating their functional interconnections was constructed with the GeneMANIA database.

### 2.5 Single-gene enrichment analysis and gene set variation analysis

To investigate the potential biological functions of core genes in the key JAK-STAT pathway, we performed Gene Set Enrichment Analysis (GSEA) and Gene Set Variation Analysis (GSVA) based on GO or KEGG pathway gene sets. GSEA used a significance threshold of P < 0.05. GSVA was performed to assess internal samples pathway-level changes using KEGG gene sets with the significance criterion (P < 0.05). Results were visualized with the “enrichplot” package.

### 2.6 Immune microenvironment analysis

We used the CIBERSORT algorithm to estimate the infiltration proportions of 22 immune-cell types in the fibrotic liver immune microenvironment, visualized the results as a bar plot, and compared immune-cell differences between the Control and Fibre groups by Wilcoxon test. Subsequently, Spearman correlation analysis was employed to compute the correlation coefficients and P-values between the JAK–STAT core genes and immune-cell infiltration, and their relationships were finally visualized by a correlation heatmap and scatter plots.

### 2.7 Mendelian randomization analysis

Using CCND1 and IL7R gene expression levels as exposures, we conducted a two-sample Mendelian Randomization (MR) analysis. Single-nucleotide polymorphisms (SNPs) that showed robust associations with expression in public eQTL datasets were selected as instrumental variables to estimate the causal effect on risk of hepatic fibrosis. Prior to analysis, only SNPs with concordant effect directions were retained, and the `harmonise_data` function was applied to align effect alleles between exposure and outcome, ensuring comparable causal estimates. Outcome data were obtained from the Finnish R12 database, covering three hepatic fibrosis–related endpoints: FIBROLIV, K11_FIBROCHIRLIV, and CHIRHEP_NAS. Inverse-variance weighted (IVW) regression served as the primary MR method, supplemented by MR-Egger, weighted median, and weighted mode estimators. Pleiotropy (MR-Egger intercept p > 0.05), heterogeneity (Cochran’s Q p > 0.05), and leave-one-out sensitivity analyses were used to assess robustness. SNPs were considered valid when IVW p < 0.05, effect directions were consistent across all four MR methods, and the pleiotropy test was non-significant. Genes meeting these criteria were further visualized with scatter plots, forest plots, funnel plots, and leave-one-out plots to present causal estimates and confirm stability.

### 2.8 Construct a miRNA-mRNA-TF interaction network

Using NetworkAnalyst 3.0 (https://www.networkanalyst.ca) to obtain interaction data: input filtered mRNAs into miRTarBase (https://mirtarbase.cuhk.edu.cn) for miRNA-mRNA targets and JASPAR (https://jaspar.genereg.net) via “TF-mRNA binding prediction” for TF-mRNA info, export miRNA-mRNA/TF-mRNA pairs; import these as edges and filtered mRNAs/miRNAs/TFs as nodes into Cytoscape (https://cytoscape.org) to build the network.

### 2.9 Single-cell sequencing analysis

The single-cell RNA sequencing dataset GSE186343 related to HBV-associated fibrosis was downloaded from the GEO database (https://www.ncbi.nlm.nih.gov/gds/). In this study, the “Seurat” package was used to process single-cell data, and only cells with a mitochondrial gene ratio < 25%, nFeature_RNA > 200 & nFeature_RNA < 7000, and nCount_RNA < 30000 were retained. The number of highly variable feature genes was set to 2,000, and then the “Harmony” package was used to integrate 6 samples. t-SNE and PCA algorithms were employed for dimensionality reduction, appropriate dimensions were selected for cell clustering, and cell populations were annotated based on the TOP5 markers of each cell cluster, with visualization by t-SNE. Subsequently, violin plots and bubble plots were used to show the expression levels of CCND1 and IL7R in different cell types, and the “FeaturePlot” function was used to present the distribution of genes in cells. Finally, the “Monocle” package was utilized to perform pseudotime trajectory analysis to explore the differentiation dynamics of different cell subpopulations. The count matrix of the Seurat object was converted into a sparse matrix, and a CellDataSet (Mono.cds) was constructed by combining gene annotations and cell metadata. After estimating size factors and dispersions, highly dispersed genes with an average expression level ≥ 0.1 were selected as ordering genes. The DDRTree method was used for dimensionality reduction, and orderCells was called to complete the pseudotime ordering of cells. plot_cell_trajectory was used to color the trajectory by cell type and pseudotime value respectively, with faceted display combined with sample origin and grading; further, the log-transformed expression levels of CCND1 and IL7R genes were integrated to visualize their expression patterns along the trajectory, revealing the temporal regulatory characteristics of genes during cell fate transition.

### 2.10 Western blot and qPCR

To validate core-gene expression, hepatitis B virus (HBV)-related fibrotic and non-fibrotic models were established in HepG2-NTCP cells with three biological replicates per group. Briefly, cells were infected with HBV at a multiplicity of infection (MOI) of 500 viral genome equivalents (vge)/cell for 24 h. After medium replacement with fresh maintenance medium, the fibrotic group was stimulated with 5 ng/mL transforming growth factor-β1 (TGF-β1) for an additional 72 h, while the non-fibrotic control group was cultured under the same conditions without TGF-β1 treatment. Cells from both groups were collected for subsequent Western blot (WB) and quantitative real-time PCR (qPCR) assays. For Western blot (WB), logarithmic-phase HepG2-NTCP cells were rinsed 2–3 times with TBS, and total protein was extracted using RIPA lysis buffer supplemented with protease inhibitors. Protein concentration was quantified via BCA assay. Equal protein amounts were separated by SDS-PAGE (75V for stacking gel, 100V for resolving gel) and transferred onto PVDF membranes (0.45μm/0.22μm based on molecular weight) via wet transfer (200mA for 1h). Membranes were blocked with 5% non-fat milk for 1h at room temperature, incubated with primary antibodies (in 5% non-fat milk/BSA) overnight at 4 °C, washed 3 times with TBST (5min each), and incubated with HRP-conjugated secondary antibodies (1:5000 dilution) for 30min at room temperature. After additional TBST washes, bands were visualized with ECL (3-10min exposure after 30s preview) and semi-quantified using Image-Pro Plus software. For quantitative real-time PCR (qPCR), total RNA was extracted by Trizol method, with purity verified by OD260/OD280 ratio (1.8-2.0). cDNA was synthesized via reverse transcription (50 °C for 15min, 85 °C for 5s) using HiScript III All-in-one RT SuperMix. qPCR was performed with 2× ChamQ SYBR qPCR Master Mix in a 20μL system (0.4μL each primer, 2μL cDNA). The program included 95 °C pre-denaturation for 5min, 40 cycles of 95 °C denaturation (10s) and 60 °C annealing/extension (30s), followed by melting curve analysis. β-actin served as the internal reference, and relative mRNA levels were calculated by the comparative Ct method. All experiments were independently repeated 3 times for reproducibility.

## 3 Results

### 3.1 Identifying the DEGs and enrichment analysis

**Figure 1:**
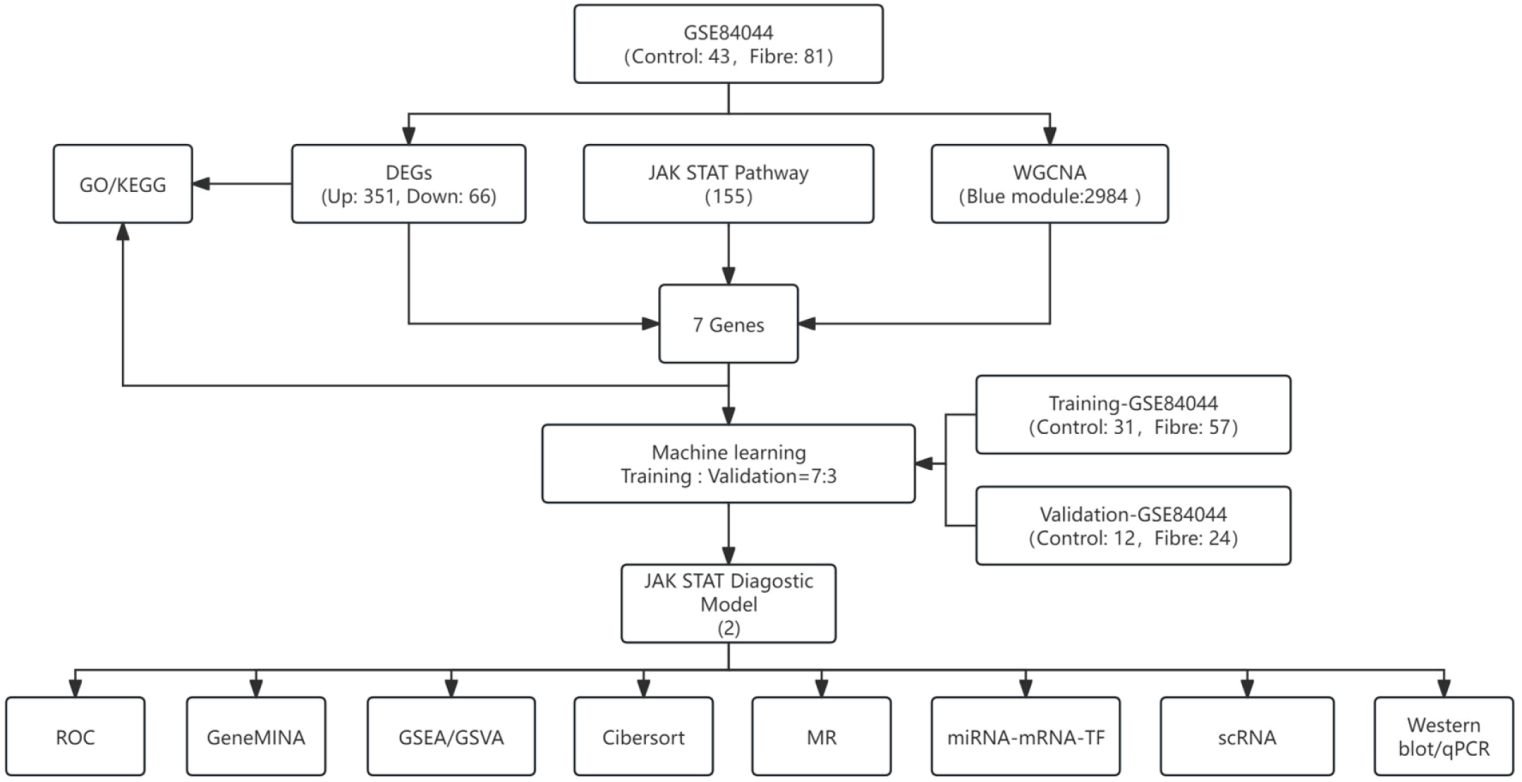
The flowchart of this research.

**Figure 2:**
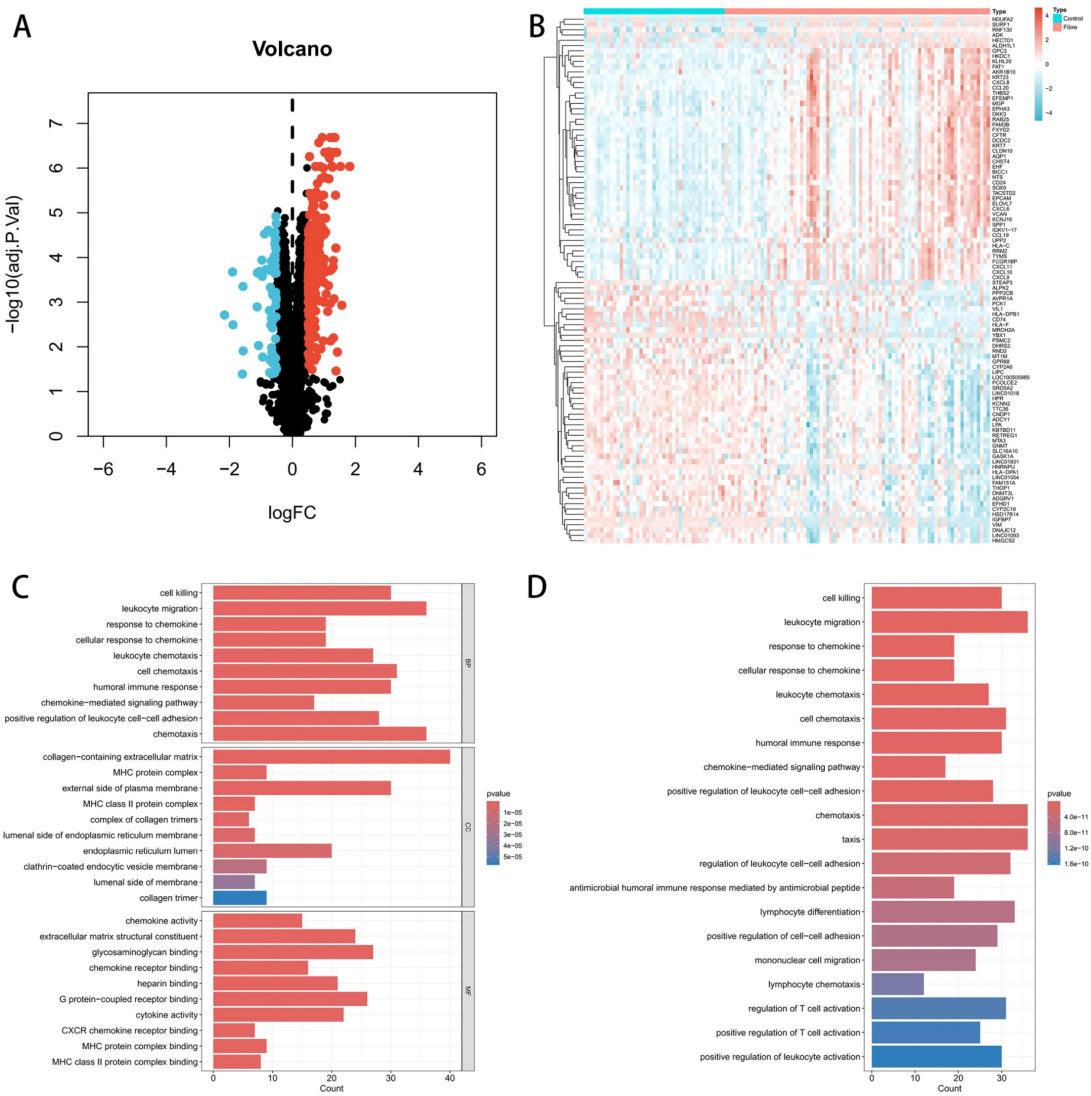
Identification of DEGs and the pathways. **(A)** The volcano plot of DEGs. **(B)** The heatmap of DEGs. **(C)** The barplot of GO enrichment analysis (biological processes, cellular components, molecular functions). **(D)** The barplot of KEGG enrichment analysis (signaling pathways and biological cascades).

The flowchart of this study could be found in **Figure 1**. By setting the standard at adjusted P-value ≤ 0.05 and |log2 fold change (FC)| ≥ 0.5, a total of 417 DEGs were identified between Fibre and Control groups including 351 upregulated and 66 downregulated genes. The result was illustrated in a volcano and heatmap plots (**Figures 2A, B**). GO enrichment analysis of the 351 DEGs focused on biological processes and cellular components, and revealed their significant involvement in the functional regulation of cell killing, leukocyte migration, chemotaxis, as well as the structural association with collagen-containing extracellular matrix (**Figure 2C**). KEGG pathway enrichment analysis further elucidated that these DEGs were mainly enriched in integrated signaling pathways and biological cascades that mediate cell killing, leukocyte migration and chemotaxis, reflecting the coordinated participation of these genes in the pathological signaling network of HBV-related liver fibrosis (**Figure 2D**).

### 3.2 Weighted gene co-expression network analysis

**Figure 3:**
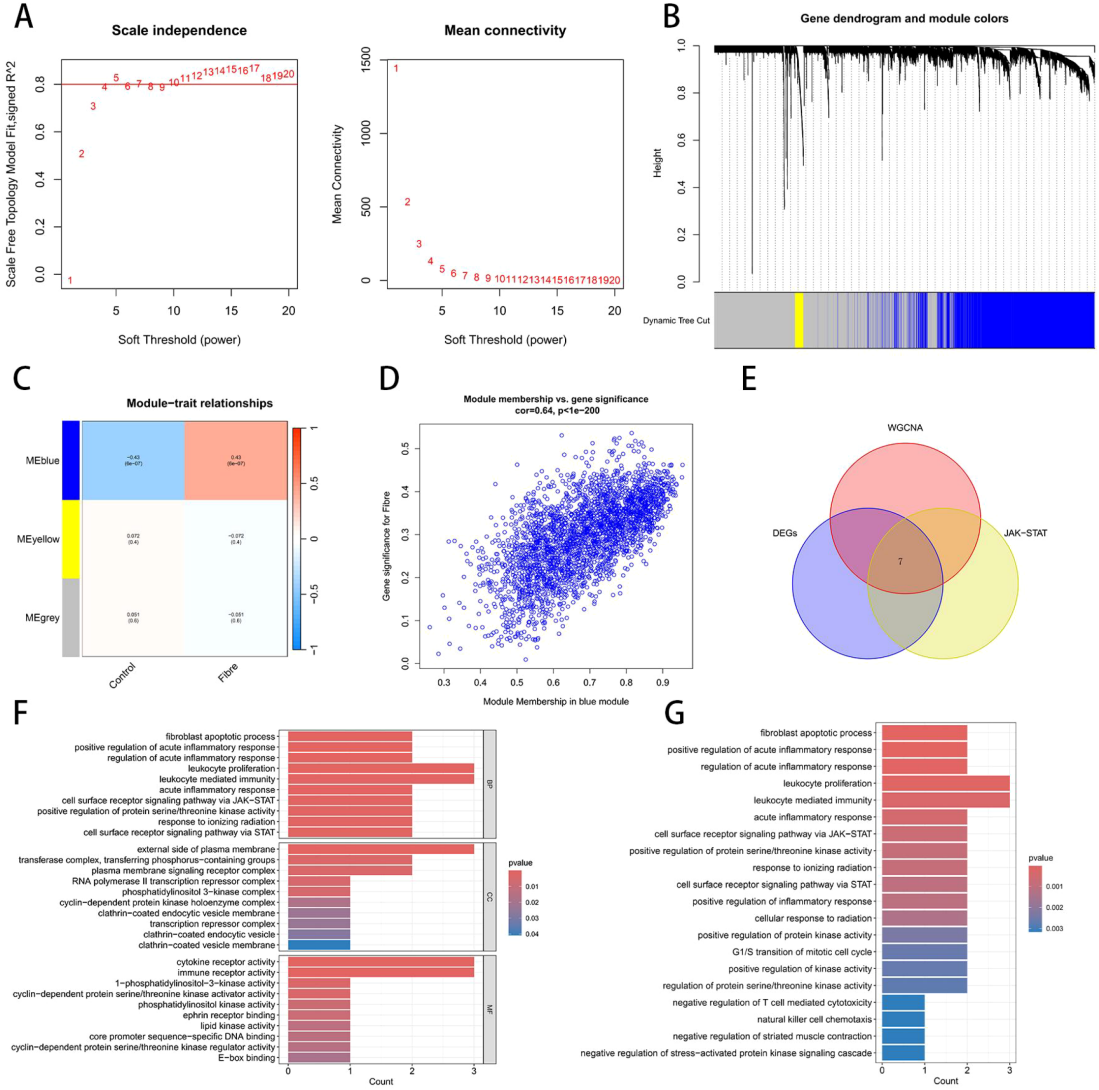
WGCNA identify the key genes. **(A)** Scale-Free Topology Fit Index For Various Soft Thresholding Powers. **(B)** Gene clustering tree and module distribution. **(C)** Heatmap of module–trait relationships. **(D)** Correlation plot between module eigengenes and phenotypes. **(E)** VENN plot showed the overlap among WGCNA, DEGs and JAK STAT. **(F)** The barplot of Go enrichment analysis. **(G)** The barplot of KEGG enrichment analysis.

WGCNA was performed to identify the Fibre related genes. When the soft-threshold power reached 13 and the R²exceeded 0.8, illustrated the WGCNA result was the most reliable (**Figure 3A**). In the co-expression analysis, all genes could be grouped into three distinct modules. In the Fibre group, the expression of the blue module was positively correlated with liver fibrosis severity (R = 0.43, p < 0.001), implying a pro-fibrotic role, whereas in the Control group it was negatively correlated with the absence of fibrosis (R = 0.43, p < 0.001), suggesting a potential protective effect under healthy conditions (**Figures 3B, C**). The blue module comprises 2,984 genes whose module membership (MM) and gene significance (GS) are highly correlated (R = 0.64, p < 0.001), confirming it as a key gene set intimately associated with liver fibrosis (**Figure 3D**). Intersection of the 2,984 module genes with 417 differentially expressed genes (DEGs) and 155 JAK–STAT pathway genes yielded seven candidate genes (**Figure 3E**). GO enrichment analysis revealed that these candidates are significantly involved in leukocyte proliferation, leukocyte-mediated immunity, the external side of the plasma membrane, and regulation of the cell-surface receptor JAK–STAT cascade (**Figure 3F**). KEGG pathway analysis further linked them to fibroblast apoptosis, acute inflammatory response, and leukocyte proliferation (**Figure 3G**).

### 3.3 Identifying the JAK STAT core genes and construct a prognostic model through machine learning

**Figure 4:**
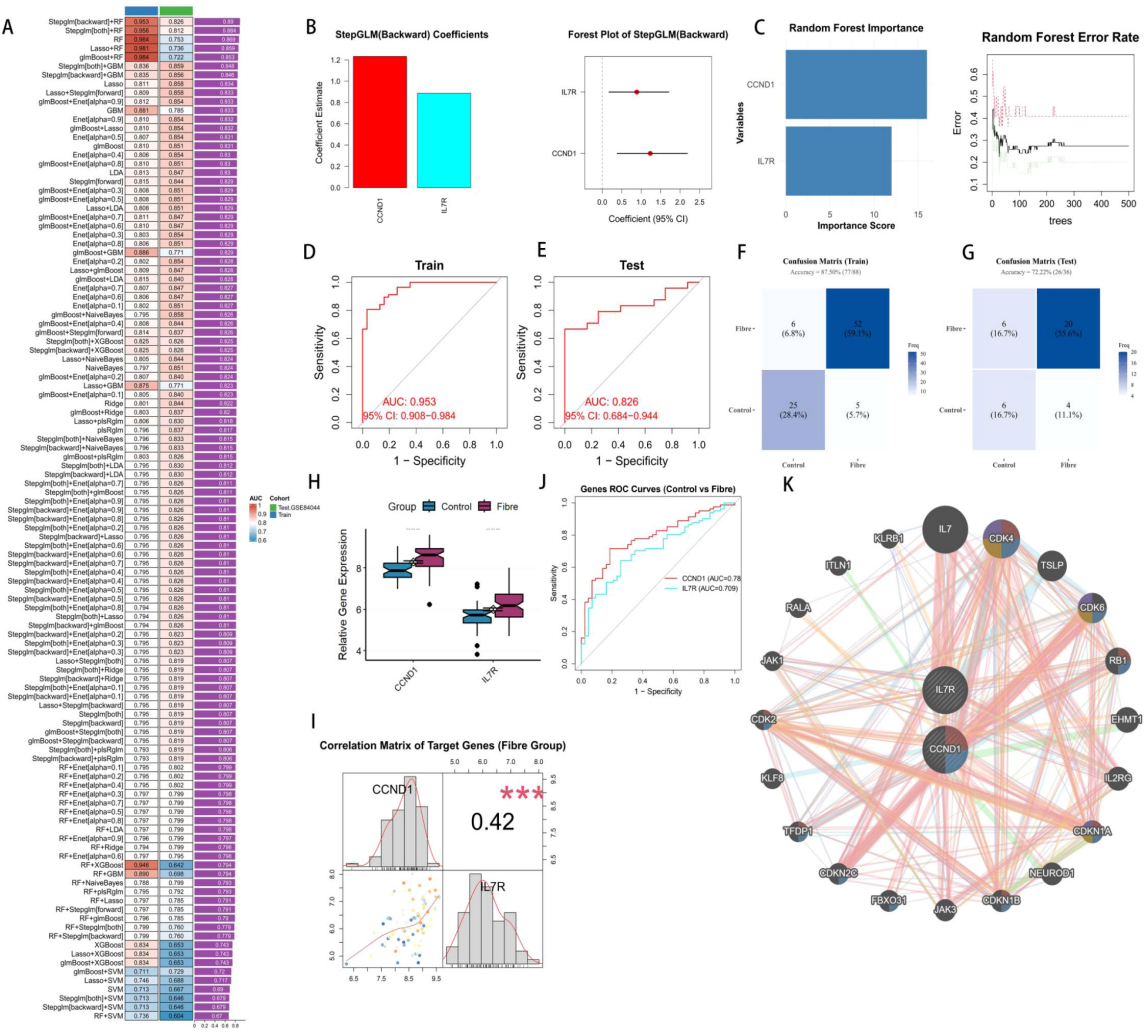
Machine learning identify the core JAK STAT genes. **(A)** The AUC heatmap of the 113 kinds of machine learning combinations. **(B)** The result of Stepglm[backward] algorithm. **(C)** The result of Random Forest algorithm. **(D, E)** ROC curves of the selected combination in training dataset and internal validation dataset. **(F, G)** The confusion matrix showed the accuracy of the selected combination in training dataset and internal validation dataset. **(H)** The expression boxplot of the core genes between Fibre and Control groups. **(I)** Correlation matrix showed the relationship between CCND1 and IL7R. **(J)** ROC curves of the JAK STAT core genes. **(K)** The correlation interaction network between CCND1 and IL7R.

A multi-level machine learning framework incorporating 113 algorithm combinations was established using 7 JAK-STAT genes. Its predictive performance was evaluated in the training set (Train-GSE84044) and internal validation set (Test-GSE84044), with the results visualized via an AUC heatmap (**Figure 4A**). Ultimately, we selected the combination of Stepglm [backward] and RF, which exhibited the highest AUC value, as our diagnostic model (**Figures 4B, C**). This combination demonstrated stable and robust performance in both the training cohort (AUC = 0.953) and the internal validation cohort GSE84044 (AUC = 0.826), with an average AUC of 0.890 (**Figures 4D, E**). Confusion matrices indicated that the diagnostic model achieved high diagnostic performance in both the training set and validation set (**Figures 4F, G**). The optimal diagnostic model comprises two core genes: CCND1 and IL7R. Both genes were significantly upregulated in HBV-related liver fibrosis samples within the total GSE84044 dataset, highlighting their functional relevance in HBV-related liver fibrosis (**Figure 4H**). The correlation density matrix revealed a positive correlation between CCND1 and IL7R (r > 0.4), suggesting that they may exert a synergistic effect in the progression of HBV-related liver fibrosis (**Figure 4I**). Single-gene ROC analysis showed that both CCND1 and IL7R had an AUC > 0.70, demonstrating favorable diagnostic performance (**Figure 4J**). Using the GeneMANIA database, we identified a complex interaction between CCND1 and IL7R, indicating their synergistic promotional role in HBV-related liver fibrosis (**Figure 4K**).

### 3.4 Exploring the potential function of CCND1 and IL7R

**Figure 5:**
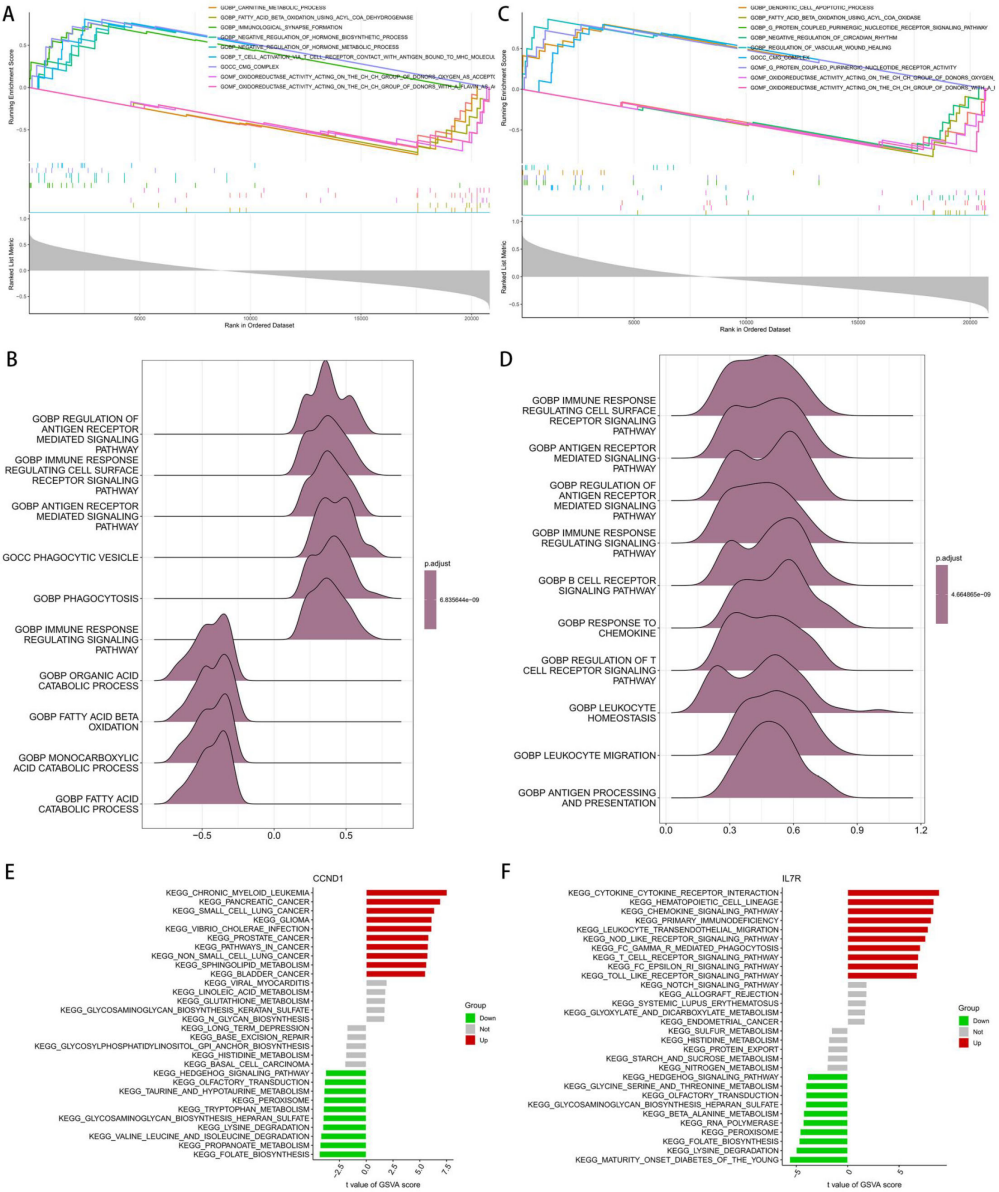
GSEA and GSVA enrichment analysis. **(A, B)** GSEA results of CCND1. **(C, D)** GSEA results of IL7R. **(E)** GSVA results of CCND1. **(F)** GSVA results of IL7R.

GSEA analysis was performed to explore the potential functions of CCND1 and IL7R in HBV-related liver fibrosis (**Figures 5A–D**). For CCND1, enrichment was observed in functional pathways including regulation of antigen receptor-mediated signaling pathway, regulation of immune response to cell surface receptor signaling pathway, phagocytic vesicle (cellular component), T cell migration, and response to chemokine (**Figures 5A, B**). As for IL7R, it was mainly enriched in immune regulatory pathways such as regulation of immune response to cell surface receptor signaling pathway, regulation of antigen receptor-mediated signaling pathway, antigen receptor-mediated signaling pathway, B cell receptor signaling pathway, regulation of immune response to signaling pathway, and regulation of T cell receptor signaling pathway (**Figures 5C, D**). HBV-related liver fibrosis samples were divided into high and low expression subgroups, and GSVA was further applied to evaluate the enrichment of functional pathways of CCND1 and IL7R between subgroups (**Figures 5E, F**). The CCND1 high-expression group was enriched in cancer-related pathways, including chronic myeloid leukemia, pancreatic cancer, small cell lung cancer, glioma, Toll-like receptor signaling pathway, and pathways in cancer; while the low-expression group was enriched in multiple metabolic pathways, such as folate biosynthesis, propanoate metabolism, valine, leucine and isoleucine degradation, glycosaminoglycan degradation, and peroxisome pathway (**Figure 5E**). The IL7R high-expression group was enriched in immune response-related pathways, such as cytokine-cytokine receptor interaction, hematopoietic cell lineage, leukocyte transendothelial migration, phagosome, T cell receptor signaling pathway, and B cell receptor signaling pathway; the low-expression group was enriched in metabolism-related pathways, including maturity-onset diabetes of the young, pyruvate metabolism, β-alanine metabolism, tryptophan metabolism, and glycine, serine and threonine metabolism (**Figure 5F**).

### 3.5 The immune microenvironment

**Figure 6:**
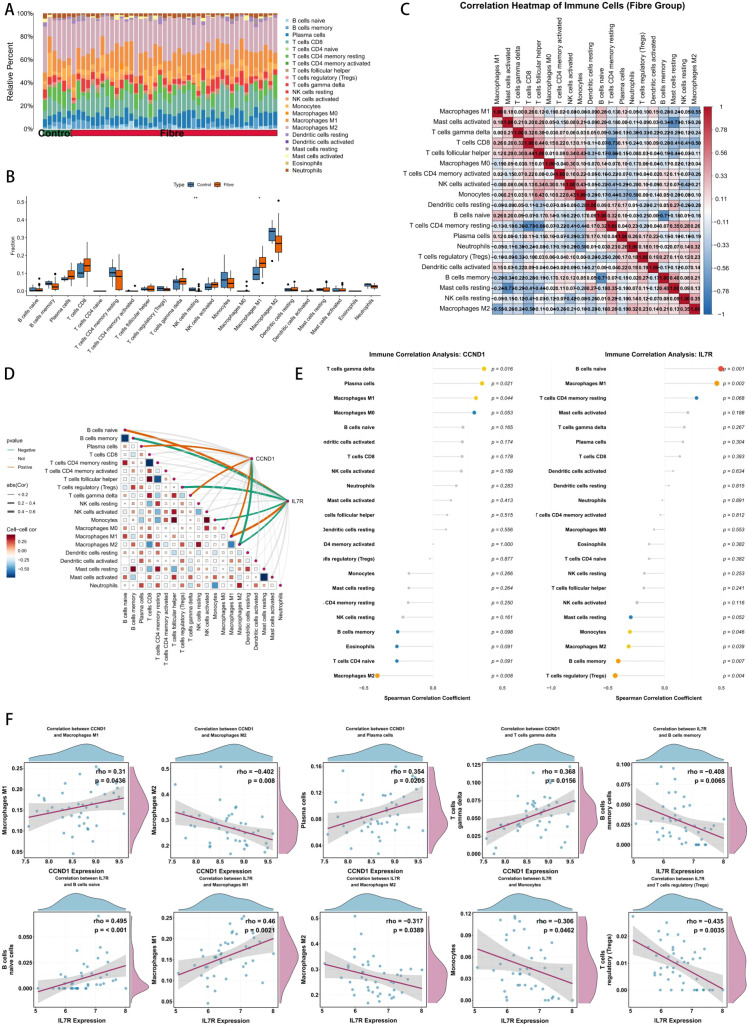
Immune infiltration analysis. **(A, B)** The difference of immune cell infiltration between Control and Fibre groups. **(C)** The heatmap showed the correlation among immune cells. **(D)** The butterfly plot showed the correlation between JAK STAT core genes and immune cells. **(E, F)** The correlation between the expression level of JAK STAT core genes and immune cells.

The CIBERSORT method was used to assess differences in the immune microenvironment between the Control and Fibre groups. We compared the immune cell composition between the Control and Fibre groups (**Figure 6A**). T cells and macrophages accounted for the major proportion in all samples, indicating their dominant role in the immune microenvironment of hepatitis B patients. Compared with the Control group, the Fibre group showed differences in the immune microenvironment: M1 macrophages were significantly increased (p < 0.05), while resting NK cells were significantly decreased, suggesting the complexity of the immune microenvironment (**Figure 6B**). The Spearman correlation heatmap indicated distinct positive and negative correlations between different immune cell types. Notably, M1 macrophages were strongly negatively correlated with M2 macrophages (r = -0.55); activated mast cells were strongly negatively correlated with resting mast cells (r = -0.73); CD8 T cells were strongly negatively correlated with CD4 memory resting T cells (r = -0.73); and naive B cells were strongly negatively correlated with memory B cells (r = -0.71). These results suggest potential mutual inhibition or phenotypic transition regulatory relationships between cell subsets with opposite functional states or different phenotypes. In contrast, follicular helper T cells were positively correlated with monocytes (r = 0.43) and activated NK cells (r = 0.34), and monocytes were also positively correlated with activated NK cells (r = 0.43), implying possible synergistic effects of these cells during immune responses (**Figure 6C**). Correlation analysis between the 2 core genes and 22 immune cell subsets showed that CCND1 was positively correlated with plasma cells, gamma delta T cells, and M1 macrophages; IL7R was positively correlated with naive B cells and M1 macrophages (**Figure 6D**). Correlation analysis between core gene expression and immune cells indicated that CCND1 expression was positively correlated with M1 macrophages, plasma cells, and gamma delta T cells (maximum r = 0.368), while negatively correlated with M2 macrophages (r = -0.402); IL7R expression was positively correlated with naive B cells and M1 macrophages (maximum r = 0.495), while negatively correlated with memory B cells, M2 macrophages, and monocytes (minimum r = -0.435). These findings highlight the diametrically opposite regulatory biases of the two genes in the immune microenvironment (**Figures 6E, F**).

### 3.6 Mendelian randomization analysis

**Figure 7:**
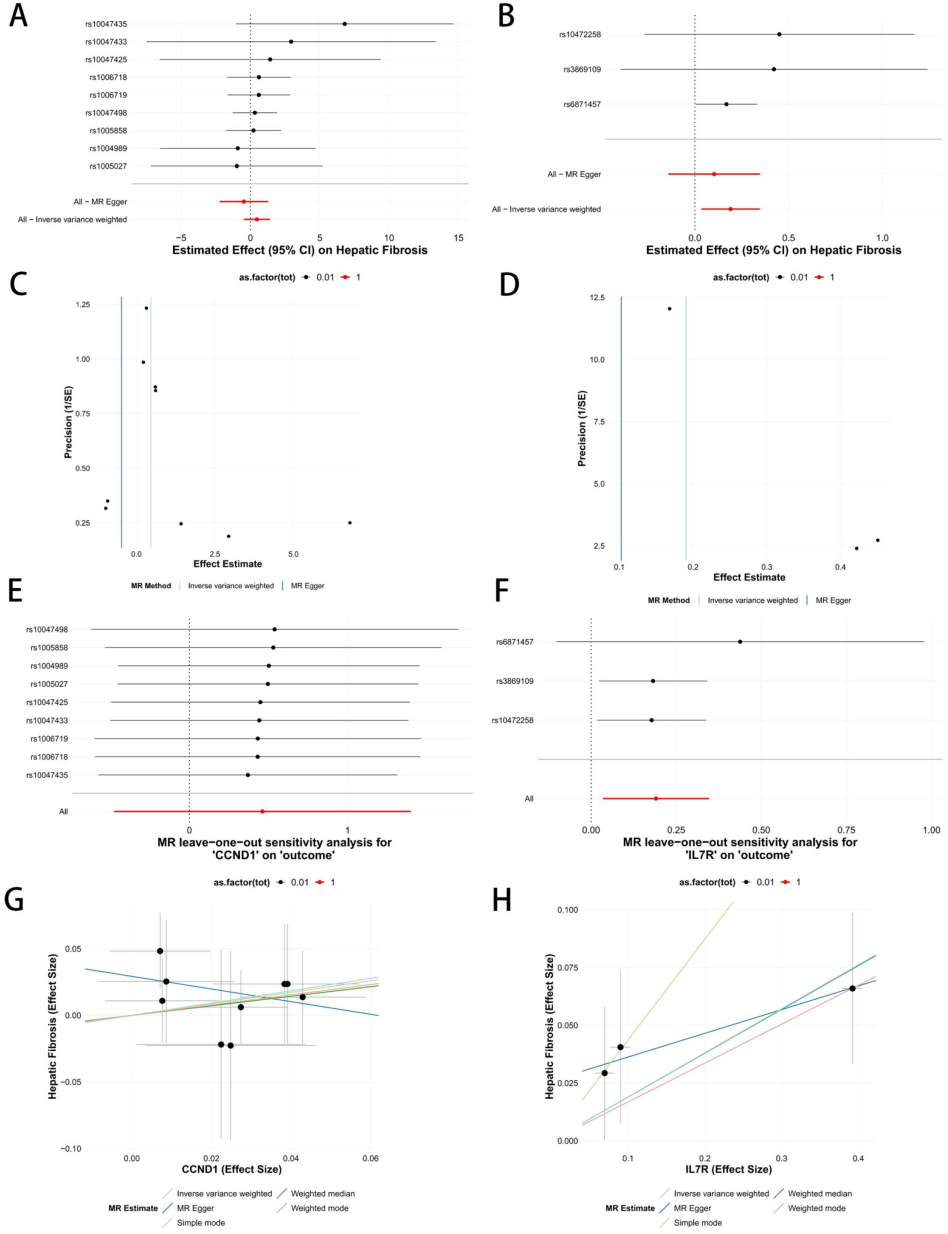
Potential causal effects of CCND1 and IL7R on hepatic fibrosis risk. **(A, B)** Forest plots present SNP-specific and pooled effect estimates (β, 95% CI) derived from MR-Egger and IVW models. **(C, D)** Funnel plots examine directional pleiotropy and between-SNP heterogeneity. **(E, F)** Leave-one-out sensitivity curves illustrate how each single SNP influences the overall estimate. **(G, H)** Scatterplots display SNP effects on exposure (gene expression) versus outcome (hepatic fibrosis risk), with regression slopes from five MR methods overlaid for comparison.

In the Finnish finngen_R12_K11_FIBROCHIRLIV_full_outcome dataset, we selected 9 SNPs strongly associated with CCND1 expression as instrumental variables. Inverse-variance weighted (IVW) Mendelian Randomization (MR) analysis showed a positive causal association between CCND1 and liver fibrosis (OR = 0.62, 95% CI: 0.11–3.59), but this did not reach statistical significance. The weighted median method yielded a consistent result (OR = 1.43, 95% CI: 0.34–6.12), also not statistically significant. MR-Egger regression showed an effect in the opposite direction, but again without statistical significance. Both the weighted mode and simple mode methods indicated a positive causal relationship between CCND1 and liver fibrosis, though not statistically significant (**Figures 7A, C, E, G**). These results suggest that CCND1 is positively associated with liver fibrosis, implying a promoting effect, but the relationship is not significant.

Similarly, in the same Finnish dataset, we selected 3 SNPs strongly associated with IL7R expression as instrumental variables. IVW MR analysis revealed a significant positive causal association between IL7R and liver fibrosis (OR = 1.20, 95% CI: 1.04–1.41, p = 0.017). The weighted median analysis also supported this conclusion (OR = 1.21, 95% CI: 1.03–1.42, P = 0.023). MR-Egger regression showed a consistent direction of effect, but did not reach statistical significance. Both the weighted mode and simple mode methods indicated a positive causal relationship between IL7R and liver fibrosis, though not statistically significant (**Figures 7B, D, F, H**).

### 3.7 Construction of a miRNA-mRNA-TF interaction network

**Figure 8:**
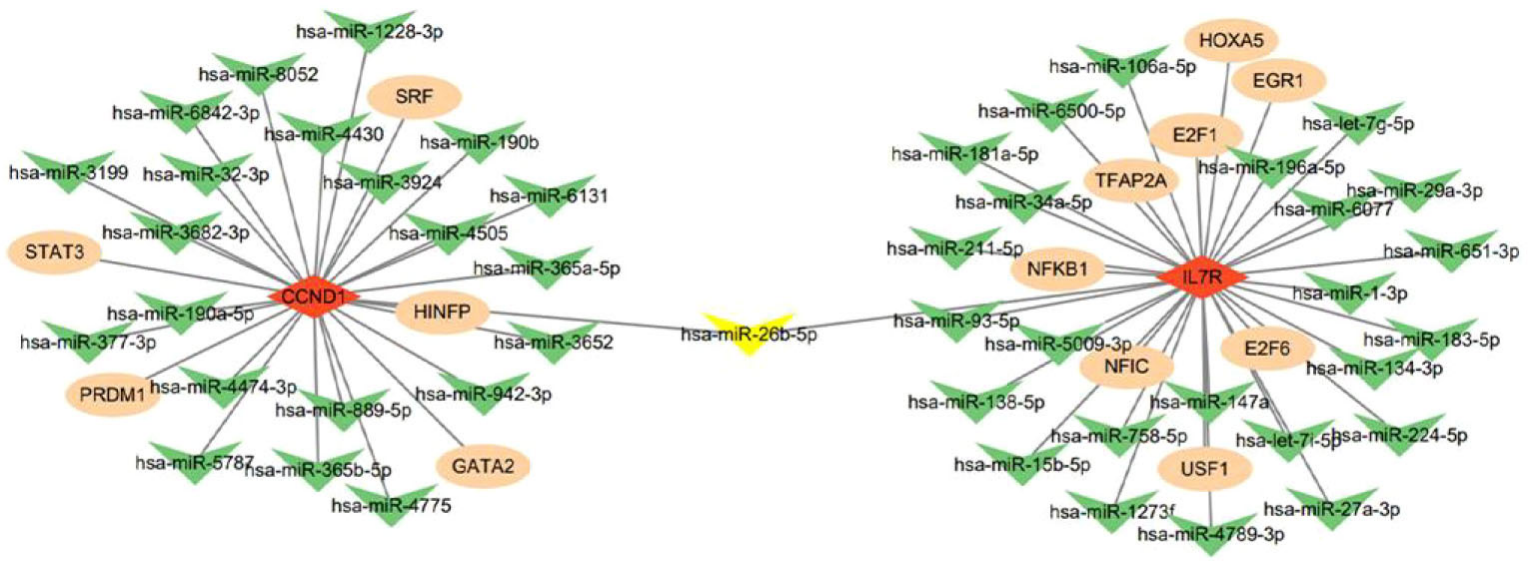
miRNA-mRNA-TF interaction network.

Recent studies have confirmed that the miRNA-mRNA-TF regulatory network plays a pivotal role in identifying core biomarkers and elucidating the molecular mechanisms underlying disease progression (20, 21). In view of this, the present study constructed this regulatory network, aiming to screen transcriptional and post-transcriptional regulators of shared biomarkers, thereby deepening the understanding of the pathophysiological processes of diseases. First, bioinformatics approaches were employed to predict miRNA-mRNA targeted interaction relationships, and a total of 47 human miRNAs were identified to target and regulate 2 core genes of the JAK/STAT pathway. Subsequently, transcription factor binding site analysis was performed, leading to the identification of 13 transcription factors that can regulate the aforementioned core genes. Finally, the interactions among the 2 JAK/STAT core genes, 47 miRNAs and 13 transcription factors were integrated, and the miRNA-mRNA-TF regulatory network was successfully constructed (**Figure 8**). Notably, the two genes CCND1 and IL7R do not share common regulatory transcription factors, but they can be synergistically regulated by the same set of miRNAs. Existing studies have demonstrated that miR-26b-5p, identified in this study, is involved in the regulation of liver fibrosis progression, and upregulating the expression level of this miRNA can significantly inhibit the progression of liver fibrosis (22, 23).

### 3.8 Single-cell sequencing analysis

**Figure 9:**
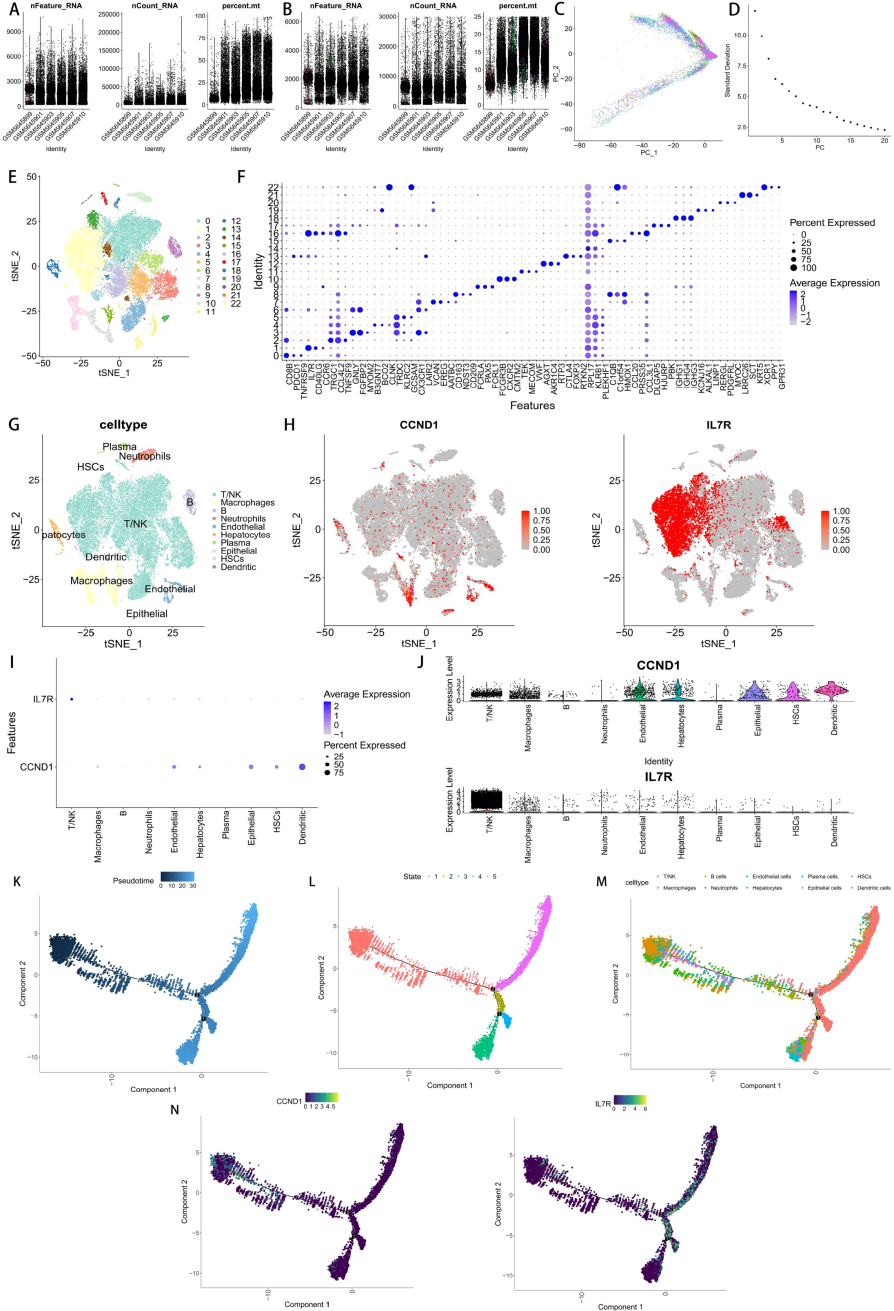
Single-cell sequencing analysis. **(A, B)** Quality control before and after (nFeature_RNA, nCount_RNA, percent.mt). **(C)** The PCA plot show the 6 samples. **(D)** Selecting the suitable Elbow for further analysis. **(E)** Cellular clustering outcomes derived from the t-SNE algorithm. **(F)** Bubble plot showed the Top 3 genes of the cell clusters. **(G)** Cellular annotation outcomes derived from the t-SNE algorithm. **(H)** The expression map of CCND1 and IL7R derived from the t-SNE algorithm. **(I, J)** Bubble plot and Violin plot showed the expression level of CCND and IL7R in different cell types. **(K-M)** Pseudotime trajectory analysis captured the differentiation dynamics across distinct cell populations, with cells ordered according to cluster identity, pseudotime progression, and developmental state. **(N)** Expression patterns of CCND1 and IL7R across the pseudotime trajectory.

Quality control was performed on the single-cell sequencing dataset (**Figures 9A, B**), followed by principal component analysis (PCA) and selection of the optimal Elbow value (**Figures 9C, D**). Finally, the t-distributed stochastic neighbor embedding (t-SNE) algorithm was used to complete cell dimensionality reduction and clustering analysis. A total of 23 cell clusters were identified using the FindClusters function, and the t-SNE plot showed their spatial distribution, with each color representing an independent cell population (**Figure 9E**). The FindAllMarkers function was used to screen for differentially expressed marker genes in each cluster, and the top 3 highly expressed marker genes in each cluster were displayed in the form of bubble plots and heatmaps (**Figure 9F**). The results of cell type annotation showed that the samples mainly contained T/NK cells, Macrophages, Endothelial cells, Hepatocytes, Neutrophils, Epithelial cells, B cells, HSCs, Dendritic cells and Plasma cells (**Figure 9G**). The visualization results of JAK-STAT core genes indicated that CCND1 was significantly upregulated in Epithelial cells, Endothelial cells, Hepatocytes, HSCs, Dendritic cells and Macrophages, while IL7R was mainly highly expressed in T/NK cells (**Figures 9H–J**). Pseudotime trajectory analysis showed that different cell clusters exhibited a clear differentiation trend along the developmental continuum (**Figures 9K–M**). The overall trajectory presented a branched tree-like structure, suggesting the existence of multiple potential differentiation paths. Cells gradually transitioned from an early state to a mature state, and the main trajectory showed the differentiation process of immune-related subsets (Macrophages, Monocytes, and Neutrophils). State-based visualization divided the cells into 5 states (State 1-5); early cells were mainly distributed in Macrophages and Neutrophils populations, while late cells were enriched in T/NK cell populations, indicating that lineage progression had a significant temporal pattern. Further analysis revealed that CCND1 expression was significantly increased in the early stage of pseudotime (corresponding to Macrophages and Neutrophils), while IL7R expression was significantly increased in the late stage of pseudotime (corresponding to T/NK cells) (**Figure 9N**).

### 3.9 Western blot and qPCR

**Figure 10:**
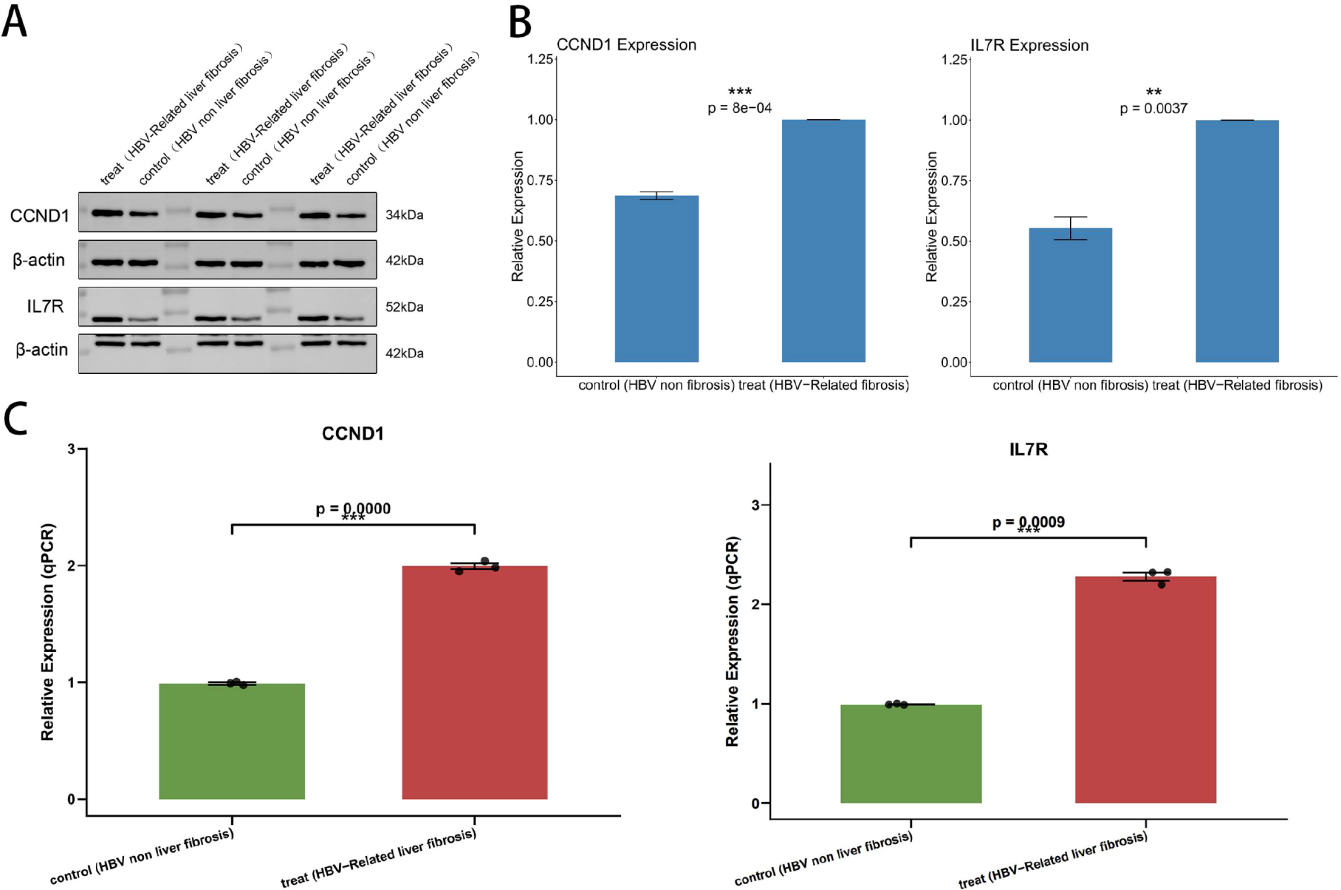
Western blot and qPCR. **(A)** The expression levels of CCND1 and IL7R were detected by Western blot, with β-actin serving as an internal control. **(B)** The bar plot showed the expression levels of CCND1 and IL7R determined by Western blot. **(C)** The bar plot showed the expression levels of CCND1 and IL7R determined by qPCR.

To verify the changes in protein expression levels of CCND1 and IL7R in hepatitis B-related liver fibrosis, we performed Western blot and qPCR analyses. The results indicated that, compared with the Control group, the protein expression levels of CCND1 and IL7R were significantly altered in the hepatitis B liver fibrosis group (Treat group). Western blot bands clearly showed that a specific CCND1 protein band (approximately 34 kD) and a specific IL7R protein band (approximately 52 kD) were detected in Treat group cells, whereas the intensity of the same bands was significantly reduced in Control group cells (**Figures 10A, B**, p<0.01). For quantitative analysis of these results, densitometric analysis was conducted on the bands from three independent experiments. The internal reference protein β-actin (approximately 42 kD) exhibited stable expression between the two groups, confirming consistent protein loading. After normalization with β-actin as the internal control, three independent qPCR replicates further confirmed that the mRNA transcription levels of CCND1 and IL7R were significantly upregulated in the Treat group (**Figure 10C**, p<0.001).

## 4 Discussion

Hepatitis B virus (HBV) infection remains a major global public health challenge, with chronic infection often progressing to liver fibrosis, cirrhosis, and even hepatocellular carcinoma (24, 25). Liver fibrosis, characterized by excessive extracellular matrix deposition and hepatic stellate cell (HSC) activation, represents a critical reversible stage in the pathological progression of chronic HBV infection (26). However, the molecular mechanisms driving HBV-related liver fibrosis are complex and not fully clarified, particularly regarding the role of key signaling pathways in coordinating inflammatory and fibrogenic processes. The JAK-STAT pathway has emerged as a central regulator of immune responses and tissue remodeling, and accumulating evidence indicates that its dysregulation is closely associated with the pathogenesis of various fibrotic diseases (13, 27). In this study, we integrated bioinformatics analyses, machine learning, single-cell sequencing and experimental validation to characterize CCND1 and IL7R as core genes within the JAK-STAT pathway that promote HBV-related liver fibrosis, thereby offering novel insights into the molecular pathogenesis and potential therapeutic targets of this disease. Single-cell sequencing analysis further refined our understanding by revealing distinct cellular localization patterns of these core genes: CCND1 was significantly upregulated in Epithelial cells, Endothelial cells, Hepatocytes, HSCs, Dendritic cells and Macrophages, while IL7R was predominantly highly expressed in T/NK cells, highlighting cell-type-specific functional contributions to fibrogenesis.

By combining differential expression analysis, weighted gene co-expression network analysis (WGCNA), and a panel of 113 machine learning algorithm combinations, we identified CCND1 and IL7R as hub genes in the JAK-STAT pathway that are associated with HBV-related liver fibrosis. Both genes were significantly upregulated in fibrotic liver tissues compared to non-fibrotic controls, and their diagnostic model achieved an AUC of 0.890, indicating strong diagnostic potential.Pseudotime trajectory analysis from single-cell data further supported their sequential involvement in fibrotic progression: CCND1 expression was prominently increased in the early pseudotime stage corresponding to macrophages and neutrophils, while IL7R expression was elevated in the late stage associated with T/NK cells, suggesting temporal coordination of their pro-fibrotic effects through the JAK-STAT pathway—with CCND1 initiating early inflammatory-fibrotic responses and IL7R amplifying late-stage immune-mediated fibrogenesis. CCND1, a cyclin family member that regulates cell cycle progression by activating cyclin-dependent kinases, has been previously implicated in various pathological processes, including cancer development and fibrotic diseases (28–30). In liver fibrosis, CCND1 overexpression has been shown to promote HSC proliferation and activation by driving cell cycle progression, thereby contributing to extracellular matrix accumulation (31). Consistent with these findings, our functional enrichment analysis revealed that CCND1 is involved in immune regulatory pathways such as antigen receptor-mediated signaling and T cell migration, as well as cancer-related pathways. This suggests that CCND1 may promote liver fibrosis through dual mechanisms: directly enhancing HSC proliferation and modulating the immune microenvironment to create a profibrotic niche.

IL7R, the receptor for interleukin-7 (IL-7) that plays a critical role in the development and survival of T and B lymphocytes, has recently attracted increasing attention due to its involvement in inflammatory and fibrotic processes (32, 33). Our results showed that IL7R is highly expressed in HBV-related liver fibrosis and exhibits a significant positive causal effect on liver fibrosis through Mendelian randomization analysis (IVW,OR = 1.20, 95% CI: 1.04–1.41, p = 0.017), highlighting its potential pathogenic role. Functional enrichment analysis demonstrated that IL7R is enriched in immune response-related pathways, including cytokine-cytokine receptor interaction, T cell receptor signaling, and B cell receptor signaling. Immune infiltration analysis further revealed that IL7R expression is positively correlated with M1 macrophages and naive B cells, while negatively correlated with M2 macrophages and memory B cells. M1 macrophages are known to secrete proinflammatory cytokines such as TNF-α and IL-6, which can activate HSCs and promote fibrogenesis (34, 35). The positive correlation between IL7R and M1 macrophages suggests that IL7R may modulate the immune microenvironment by promoting M1 macrophage polarization, thereby amplifying the inflammatory response and accelerating liver fibrosis progression (36). Additionally, IL-7/IL7R signaling has been shown to regulate T cell homeostasis and function, and its dysregulation may lead to impaired immune surveillance and persistent inflammatory responses in chronic HCV infection, further contributing to fibrotic progression (37, 38), with single-cell data providing direct evidence of IL7R’s expression in T/NK cells that mediate these immune effects.

The positive correlation between CCND1 and IL7R in HBV-related liver fibrosis suggests that these two molecules may participate in the fibrotic process through the JAK–STAT pathway. Studies have shown that CCND1 influences hepatic stellate cell (HSC) activation and promotes excessive extracellular-matrix production in liver fibrosis (29).The JAK–STAT pathway has been extensively reported to contribute to hepatic fibrosis, with cytokines such as IL-6, IL-22 and IFN-γ activating this pathway (13). Although the precise mechanisms by which IL7R operates in liver fibrosis remain to be fully defined, existing evidence indicates that it may participate in disease progression by modulating the immune microenvironment (39, 40). Single-cell sequencing further uncovered that CCND1 and IL7R are expressed in non-overlapping cell populations (epithelial/hepatocyte/macrophage vs. T/NK cells), suggesting their synergistic effects may be mediated through intercellular signaling within the fibrotic liver microenvironment rather than cell-autonomous interactions. This multi-molecular interplay underscores the complexity of the molecular networks underlying HBV-related liver fibrosis and implies that simultaneous targeting of several core genes may be required for effective therapy.

Immune infiltration analysis revealed significant alterations in the immune microenvironment of HBV-related liver fibrosis, with a marked increase in M1 macrophages and a decrease in resting NK cells in fibrotic tissues compared to non-fibrotic controls. M1 macrophages are key producers of proinflammatory cytokines and profibrotic mediators, and their accumulation is closely associated with HSC activation and extracellular matrix deposition (34, 41). Our results showed that both CCND1 and IL7R are positively correlated with M1 macrophages, suggesting that these genes may promote M1 macrophage infiltration or polarization to exacerbate liver fibrosis. Single-cell sequencing validated the presence of these immune cell subsets and their spatial distribution, confirming that the fibrotic liver microenvironment is characterized by dysregulated immune cell populations with functional implications for fibrogenesis. Additionally, we observed complex correlations between different immune cell subsets, such as the strong negative correlation between M1 and M2 macrophages, and between naive and memory B cells. These findings indicate that the immune microenvironment in HBV-related liver fibrosis is highly dysregulated, with a shift toward a proinflammatory phenotype that favors fibrogenesis. The close association between CCND1/IL7R and immune cell infiltration further supports the notion that the JAK-STAT pathway regulates liver fibrosis through immune-modulatory mechanisms.

Mendelian randomization (MR) analysis provided causal evidence for the role of IL7R in HBV-related liver fibrosis, with IVW analysis showing a significant positive causal effect (OR = 1.20, 95% CI: 1.04-1.41, p=0.017). This strengthens the reliability of our findings by minimizing confounding factors and confirming that IL7R overexpression is not merely a consequence of liver fibrosis but may actively drive its progression. In contrast, the causal effect of CCND1 on liver fibrosis did not reach statistical significance, although a positive trend was observed. This may be due to the limited number of instrumental variables or the involvement of CCND1 in multiple signaling pathways that could have opposing effects on liver fibrosis. Further studies with larger sample sizes and more robust instrumental variables are needed to confirm the causal role of CCND1(Before Revise). Notably, while IL7R demonstrated a significant causal effect on liver fibrosis in MR analysis, CCND1 showed significant differential expression and diagnostic value but lacked statistical evidence for a causal relationship, suggesting it may function as a biomarker of fibrotic progression rather than a direct causal driver.

The miRNA-mRNA-TF regulatory network constructed in this study revealed that CCND1 and IL7R can be synergistically regulated by a set of miRNAs, including miR-26b-5p, which has been previously shown to inhibit liver fibrosis progression (23, 42). This suggests that post-transcriptional regulation of CCND1 and IL7R by miRNAs may play an important role in HBV-related liver fibrosis, and targeting these regulatory axes could be a potential therapeutic strategy. For example, upregulating miR-26b-5p expression may simultaneously suppress CCND1 and IL7R expression, thereby inhibiting both HSC proliferation and proinflammatory immune responses (43, 44).

Experimental validation via Western blot and qPCR confirmed that CCND1 and IL7R are significantly upregulated at both the mRNA and protein levels in HBV-related fibrotic liver tissues, consistent with our bioinformatics results. These findings provide direct evidence for the involvement of CCND1 and IL7R in HBV-related liver fibrosis and support their potential as diagnostic biomarkers and therapeutic targets. Targeting CCND1 and IL7R may offer a dual approach to treating HBV-related liver fibrosis: inhibiting HSC activation and proliferation through CCND1 suppression, and modulating the immune microenvironment to reduce inflammation through IL7R targeting. Several small-molecule inhibitors targeting CCND1 or the JAK-STAT pathway are currently under development for cancer and inflammatory diseases, and their repurposing for liver fibrosis deserves further investigation.

Despite the significant findings of this study, several limitations should be acknowledged. First, the bioinformatics analyses were primarily based on a single GEO dataset (GSE84044), and validation with additional independent cohorts is needed to confirm the generalizability of our results. Second, although we performed experimental validation using HepG2-NTCP cells, *in vitro* cell models cannot fully recapitulate the complex *in vivo* microenvironment of HBV-related liver fibrosis. Future studies using animal models of HBV infection and liver fibrosis are required to verify the *in vivo* role of CCND1 and IL7R. Third, the specific molecular mechanisms by which CCND1 and IL7R interact with the JAK-STAT pathway to promote liver fibrosis remain unclear. Further studies are needed to investigate the downstream signaling cascades and protein-protein interactions involved. Finally, while our MR analysis provided causal evidence for IL7R, the potential confounding effects of other genetic or environmental factors cannot be completely excluded.(Before Revise) {Despite the significant findings of this study, several limitations should be acknowledged. First, the bioinformatics analyses and machine learning model construction were primarily based on a single GEO dataset (GSE84044), for which we only performed a 7:3 internal split to generate the training and validation sets without the support of an independent external validation cohort. We fully recognize this inherent limitation of single-dataset internal validation, and explicitly acknowledge that the constructed diagnostic model may have a potential risk of overfitting, which may limit the generalizability of our results to some extent. Second, although we performed experimental validation using HepG2-NTCP cells, *in vitro* cell models cannot fully recapitulate the complex *in vivo* microenvironment of HBV-related liver fibrosis. Future studies using animal models of HBV infection and liver fibrosis are required to verify the *in vivo* role of CCND1 and IL7R. Third, the specific molecular mechanisms by which CCND1 and IL7R interact with the JAK-STAT pathway to promote liver fibrosis remain unclear. Further studies are needed to investigate the downstream signaling cascades and protein-protein interactions involved. Finally, while our MR analysis provided causal evidence for IL7R, the potential confounding effects of other genetic or environmental factors cannot be completely excluded. (After Revise)}.

## 5 Conclusion

In conclusion, our study identifies CCND1 and IL7R as core genes of the JAK-STAT pathway that promote HBV-related liver fibrosis (Before Revise). In conclusion, our study identifies IL7R as a causal core gene and CCND1 as a robust diagnostic biomarker of the JAK-STAT pathway in HBV-related liver fibrosis. IL7R demonstrates a significant causal effect on liver fibrosis progression, while CCND1 exhibits strong diagnostic performance (AUC > 0.75) and differential expression but lacks evidence for a direct causal role (After Revise).These genes act synergistically to regulate the immune microenvironment and drive fibrogenesis, and their diagnostic model exhibits strong performance. IL7R shows a significant causal role in liver fibrosis, making it a particularly promising therapeutic target(Before Revise). Only IL7R shows a significant causal role in liver fibrosis, making it a particularly promising therapeutic target, whereas CCND1 may serve primarily as a diagnostic indicator and potential accessory marker of disease activity (After Revise). Our findings deepen the understanding of the molecular mechanisms underlying HBV-related liver fibrosis and provide novel biomarkers and potential therapeutic strategies for the diagnosis and treatment of this disease. Future studies should focus on developing specific inhibitors targeting CCND1 and IL7R, and exploring their efficacy in preclinical and clinical settings.

## Data Availability

Publicly available datasets were analyzed in this study. The GSE84044 and GSE186343 can be retrieved from GEO database (https://www.ncbi.nlm.nih.gov/gds/) and the outcome data of MR can be retrieved from Finnish R12 database (https://www.finngen.fi/en/access_results).
